# The optimal fluoroscopic views to rule out intra-articular screw penetration during acetabular fracture fixation

**DOI:** 10.1007/s00264-023-06002-6

**Published:** 2023-10-19

**Authors:** Aissam Elmhiregh, Ashraf T. Hantouly, Osama Alzoubi, Bivin George, Mohsen Ahmadi, Ghalib Ahmed

**Affiliations:** 1 Department of Orthopaedic Surgery, Surgical Specialty Center, Hamad Medical Corporation, Doha, Qatar; 2 Clinical Imaging Department, Hamad Medical Corporation, Doha, Qatar

**Keywords:** Acetabulum, Fracture, Fluoroscopy, Intra-articular screw, Complications

## Abstract

**Purpose:**

To determine the ideal view(s) and the minimum number of intraoperative fluoroscopic views required to rule out any intra-articular screw violation in acetabular fractures fixation.

**Methods:**

This study was conducted using a series of fluoroscopic examinations of pelvic synthetic models with screws positioned in different planes around the acetabulum. Ten screws were placed in the synthetic pelvis models in different planes of the acetabulum. Seven views were taken for each screw. Radiographic images were evaluated by 14 orthopaedic surgeons who were asked to assess joint violation and the view(s) required for assessment.

**Results:**

The observers’ accuracy rate in identifying joint violation was 82.1% for the anterior part of the anterior column and the superior part of the posterior column, 89.3% for the posterior part of the anterior column and the inferior part of the posterior column, and 92.9% for the quadrilateral plate. The sensitivity was 100% for the anterior and posterior parts of the anterior column and the inferior part of the posterior column, 87.5% for the superior part of the posterior column, and 85.7% for the quadrilateral plate. The specificity was 100% for the quadrilateral plate, 80% for the superior part of the posterior column and the posterior part of the anterior column, 78.6% for the inferior part of the posterior column, and 66.7% for the anterior part of the anterior column. There was a strong overall interobserver and intra-observer agreement with intraclass correlation coefficient (ICC) of 0.709 and 0.86, respectively.

**Conclusions:**

This study confirms the hypothesis that in a concave surface/joint fixation, such as the acetabulum, the probability of joint violation is unlikely if there is no evidence of it within a single fluoroscopic view. In acetabulum fracture fixation with a screw violating the joint, the screw’s presence was evident within the joint space in all fluoroscopic views. However, the absence of joint violation in one fluoroscopic view was adequate to rule out joint penetration.

## Introduction

Acetabulum fractures pose a significant challenge for orthopaedic surgeons due to their diverse range of injury patterns. These fractures often necessitate surgical fixation, particularly when displacement occurs or when the weight-bearing area is involved [1, 2]. However, the operative management, encompassing both open reduction and percutaneous fixation, mandates a careful intraoperative radiological evaluation using sufficient and accurate fluoroscopic views [3–8]. Despite the comprehensive literature describing the techniques of acetabular fracture fixation, there is still a lack of consensus on the optimal view(s) and minimum number of radiographic views required to rule out intra-articular screw penetration [9–13].

Therefore, this study aimed to determine the ideal number and specific fluoroscopic view(s) required to confirm the absence of intra-articular screw penetration in acetabular fracture fixation. Additionally, the study tested the hypothesis that for concave surfaces such as the acetabulum, the absence of screw penetration in a single view would suffice to eliminate concerns of joint violation.

## Materials and methods

### Study design

This experimental study was conducted to determine the minimum number and optimal intra-operative fluoroscopic view(s) required to confirm the absence of intra-articular screw penetration in acetabular fracture fixation. The study adhered to the guidelines for reporting reliability and agreement studies (GRRAS) [14].

### Study setting

This study was approved by the institutional review board (MRC-01–19) and was conducted at a level I trauma centre. Anatomical synthetic pelvic bone models were used in the study, with ten screws inserted in various locations around the acetabulum. Five of the screws were inserted with intra-articular penetration, while the other half were without. Seven pelvic fluoroscopic views were obtained for each screw to evaluate the radiographic screw position and correlate it with the actual pelvic model screw location, mimicking the intraoperative conditions during periacetabular screw insertion in acetabular fracture fixation.

**Fig. 1:**
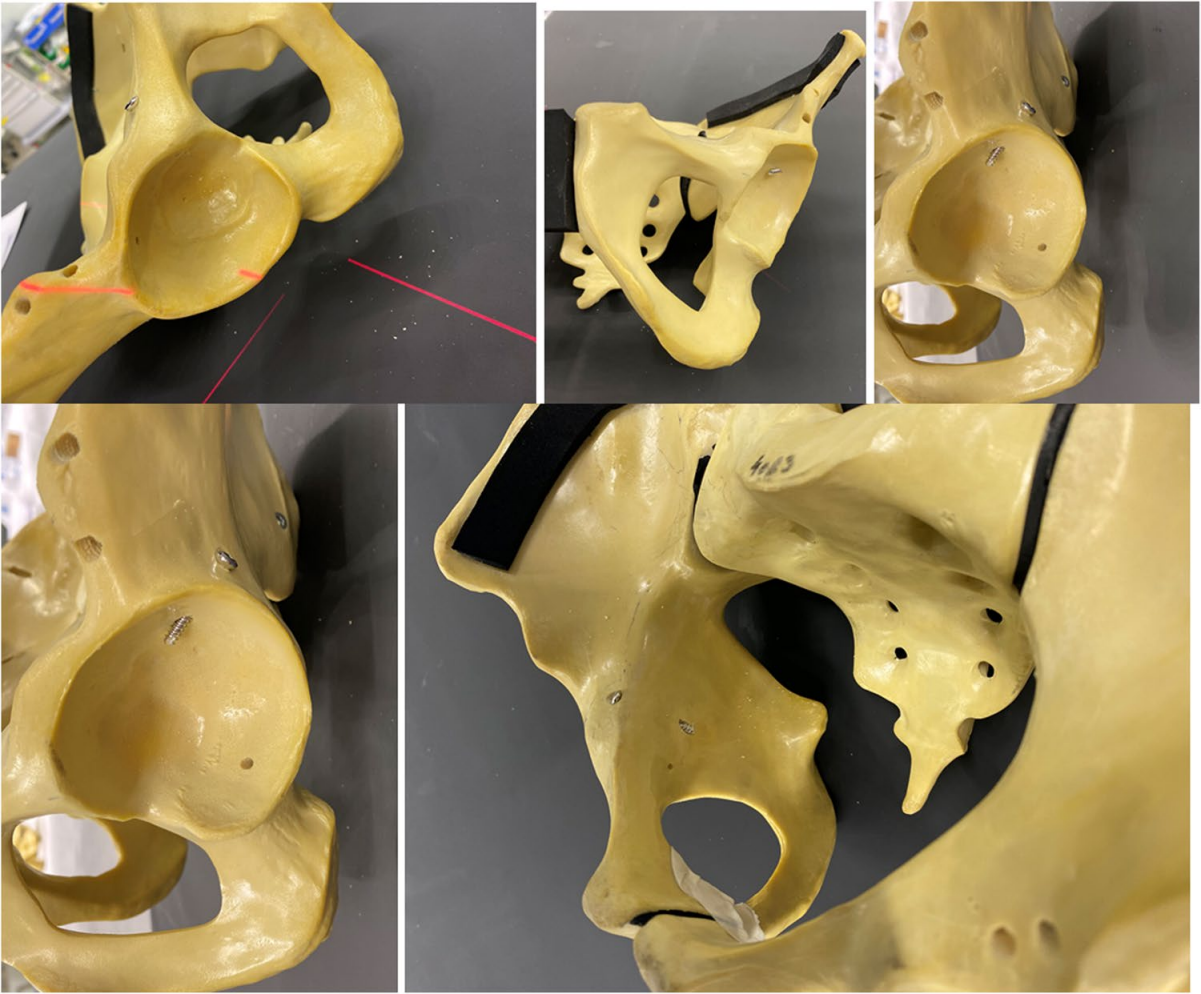
Clinical photograph of the pelvic model demonstrating different periacetabular screws with different positions and relations to the joint

In this study, a pair of identical synthetic anatomical pelvic models (as illustrated in Fig. 1) was used. Drill holes were made at different positions around the acetabulum using a 2.5-mm drill bit. This encompassed two drill holes in the anterior and posterior parts of the anterior column, two drill holes in the superior and inferior parts of the posterior column, and one drill hole in the quadrilateral plate. A total of ten 3.5-mm screws were inserted into the pre-drilled holes and labeled numerically in an ascending order (Table 1). Five screws were placed intra-articularly, while the remaining five were without intra-articular penetration. Specifically, intra-articular screws were labelled with odd numbers, whereas those without intra-articular penetration were labelled with even numbers. The locations of the screws were as follows: the anterior and posterior parts of the anterior column and the superior and inferior parts of the posterior column and the quadrilateral plate (Fig. 1).

**Table 1:** The positions of the ten periacetabular screws

Screw label	Joint violation	Screw position
1	Yes	Anterior part of anterior column
2	No	Anterior part of anterior column
3	Yes	Posterior part of anterior column
4	No	Posterior part of anterior column
5	Yes	Superior part of posterior column
6	No	Superior part of posterior column
7	Yes	Inferior part of posterior column
8	No	Inferior part of posterior column
9	Yes	Quadrilateral plate
10	No	Quadrilateral plate

**Fig. 2:**
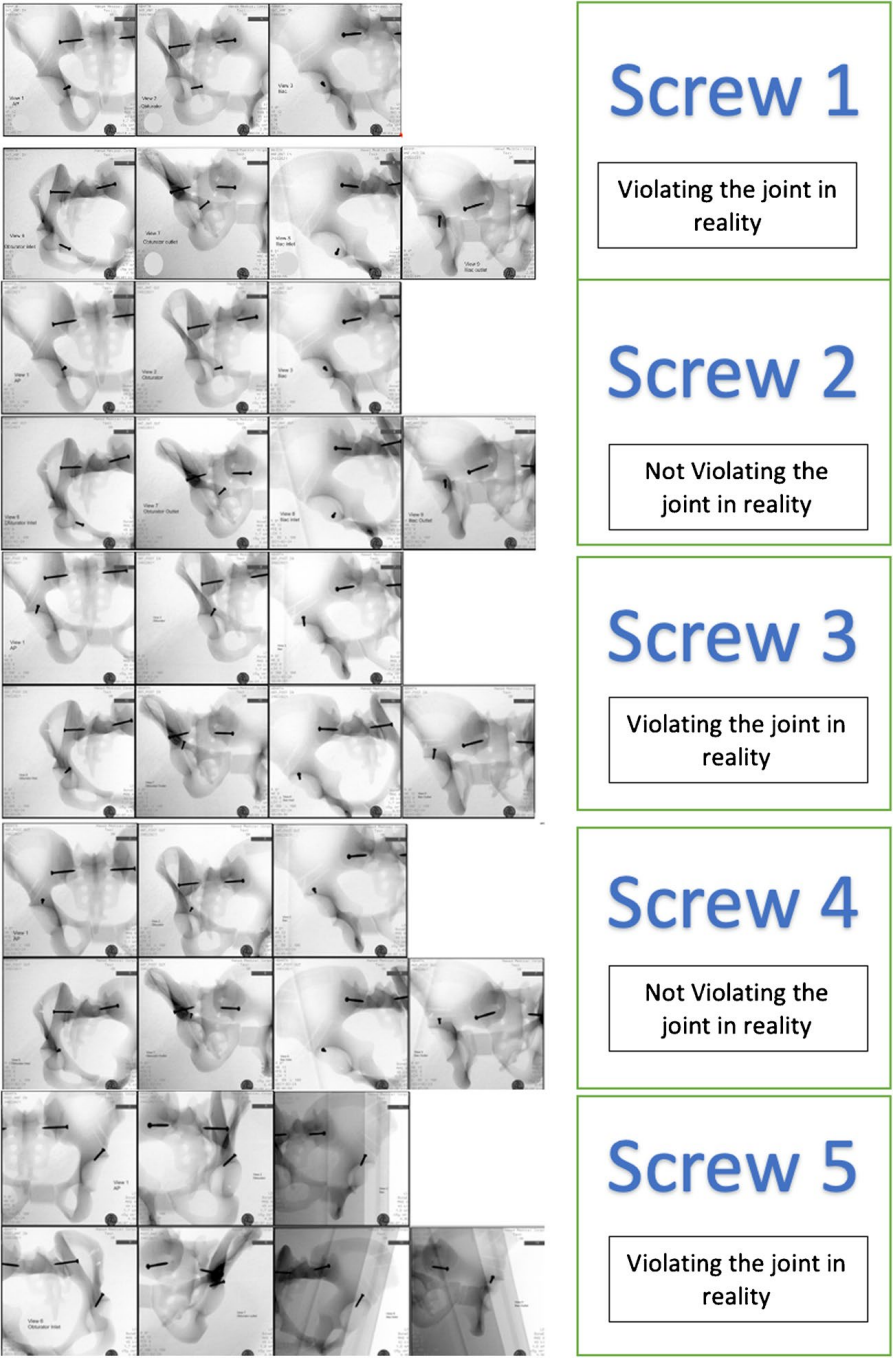

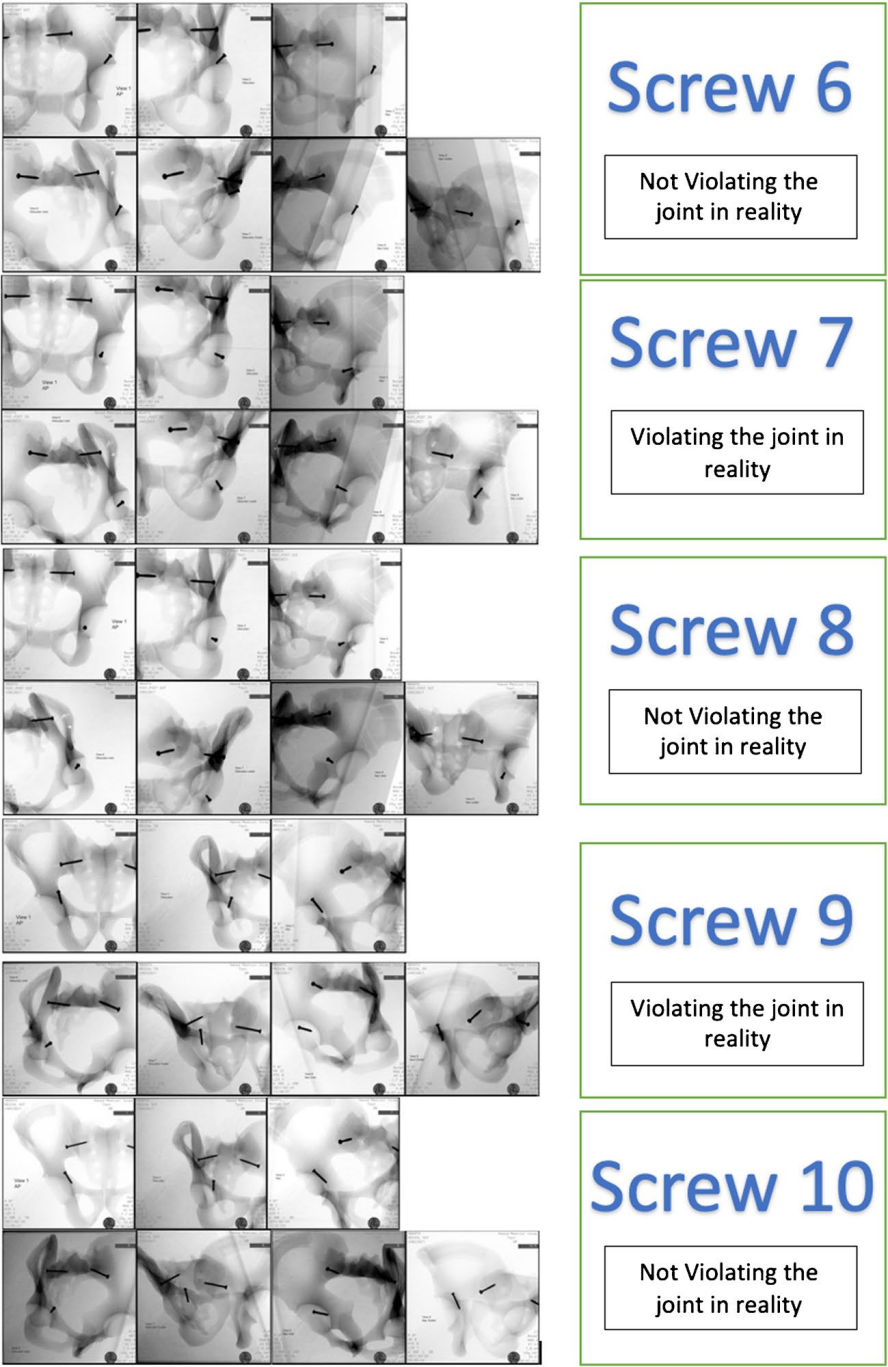
Fluoroscopy captures viewing the ten screws with seven different radiological projections; please note that the studied screws are the ones near the acetabulum, while the other two screws at the sacro-iliac joints are part of pelvic model to hold the parts of model

The synthetic pelvic models were then positioned on a radiolucent operative table and seven fluoroscopic radiographic views (anteroposterior, obturator oblique, iliac oblique, obturator inlet, obturator outlet, iliac inlet, and iliac outlet) were taken for the pelvic models using a mobile C-arm fluoroscopy machine (Fig. 2).

To ensure consistency and accuracy, all the fluoroscopic views were taken by a certified radiographic technician using a standard radiographic technique with the same machine. This was vital to maintain proper calibrations of the views’ angles. The radiographic images were saved electronically on the primary investigator’s computer, and only two authors had access to the coded data.

After preparing and coding the data, the images and questionnaires were electronically sent to a group of 14 evaluators, comprising orthopedic trauma fellows and consultants. The task was to evaluate the seven obtained fluoroscopic views for each screw to determine the presence or absence of intra-articular screw violation. This process was conducted independently and blindly, with the images and questionnaires sent electronically to each evaluator on two separate occasions, one month apart. The results were correlated with the actual position of the screws in the pelvic models to measure accuracy.

### Inclusion and exclusion criteria

Ten synthetic pelvic models were required, with screws inserted at various locations around the acetabulum and seven fluoroscopic views taken for each screw position. Incomplete questionnaires were excluded.

### Data collection

The collected baseline variables comprised the parts of the acetabulum column, screw position, and the specific fluoroscopic radiographic views. The primary outcomes were the minimum number and most effective intra-operative fluoroscopic views for identifying intra-articular screw penetration.

### Radiographic assessment

Fourteen independent observers evaluated the radiographs. Before the assessment sessions, the first and senior authors conducted a training session to all observers. The training focused on the use of the fluoroscopic radiographic views, assessment of acetabular screws, and identifying intra-articular screw violation.

The observers were instructed to evaluate the screws’ positions in the pelvic models using the fluoroscopic views. Each participating surgeon was presented with two questions for each image: (1) whether there was intra-articular screw penetration, to which the surgeon responded with a “yes” or a “no.” (2) Based on the previous question, they were asked to identify the view(s) needed to answer the first question.

After 1 month, a similar session was conducted for the same group of observers. The observers did not have access to the radiographs during the interval between the two sessions. Figure 2 displays examples of selected radiographs showcasing screws positioned in different configurations.

### Statistical analysis

The data was analyzed for accuracy, sensitivity, and specificity for radiographs in determining the presence of screw penetration and the location of screw penetration. Interobserver reliability was determined based on a Kappa (k) statistic. Sensitivity refers to the ability of a test to detect disease when it is present. Specificity is the ability of a test to detect the absence of disease when it is not present. For the purpose of this study, sensitivity refers to the proportion of the pelvic model with intra-articular screw penetration accurately identified by the radiographic views; specificity is the proportion of those without intra-articular screw penetration correctly identified. Accuracy is a summary measure of the diagnostic correctness, consisting of both the positive and negative tests that were correctly interpreted. Interobserver and intra-observer agreement on screw penetration as compared with the reality was assessed using kappa statistic or ICC which measures agreement between multiple observers, comparing actual agreement to the agreement that would be expected due to chance. The interrater and intra-rater reliability analysis using the Kappa statistic was performed to determine consistency among reviewers about joint violation in screw position. The 95% confidence intervals were calculated using the following formula: Estimate + / − 1.96 SE. Intraclass correlation coefficient (ICC) single rating two-way random effect model was applied to find the overall interrater and intra-rater agreement. Statistical calculations were performed using SPSS version 28.0 (IBM Corp., Armonk, NY, USA) and MedCalc Software Ltd, Ostend, Belgium (for Sensitivity, Specificity, PPV, NPV).

## Results

### Characteristics of pelvic models and observers’ responses

A pair of identical synthetic pelvic models were used with ten screws inserted in various locations around the acetabulum. Among these screws, five were placed violating the joint, while the other five were placed in the acetabulum avoiding joint penetration. The details of the pelvic models and radiographic views are presented in Table 1 and Figs. 1 and 2.

A total of 140 responses were collected for each pelvic view of all screws, comprising 70 responses for the extra-articular screws and 70 for the intra-articular ones. These responses were obtained from 14 orthopaedic trauma surgeons, each of whom evaluated 70 images.

### Extra-articular screw position

Regarding the extra-articular screws, the observers exhibited an overall accuracy rate of 91.4% (*n* = 64). Screw number 8 had an accuracy rate of 100%, while screws number 2, 4, and 10 had an accuracy rate of 92.85%. The lowest accuracy rate was recorded for screw number 6, with a rate of 85.7% (see Table 2).

**Table 2:**
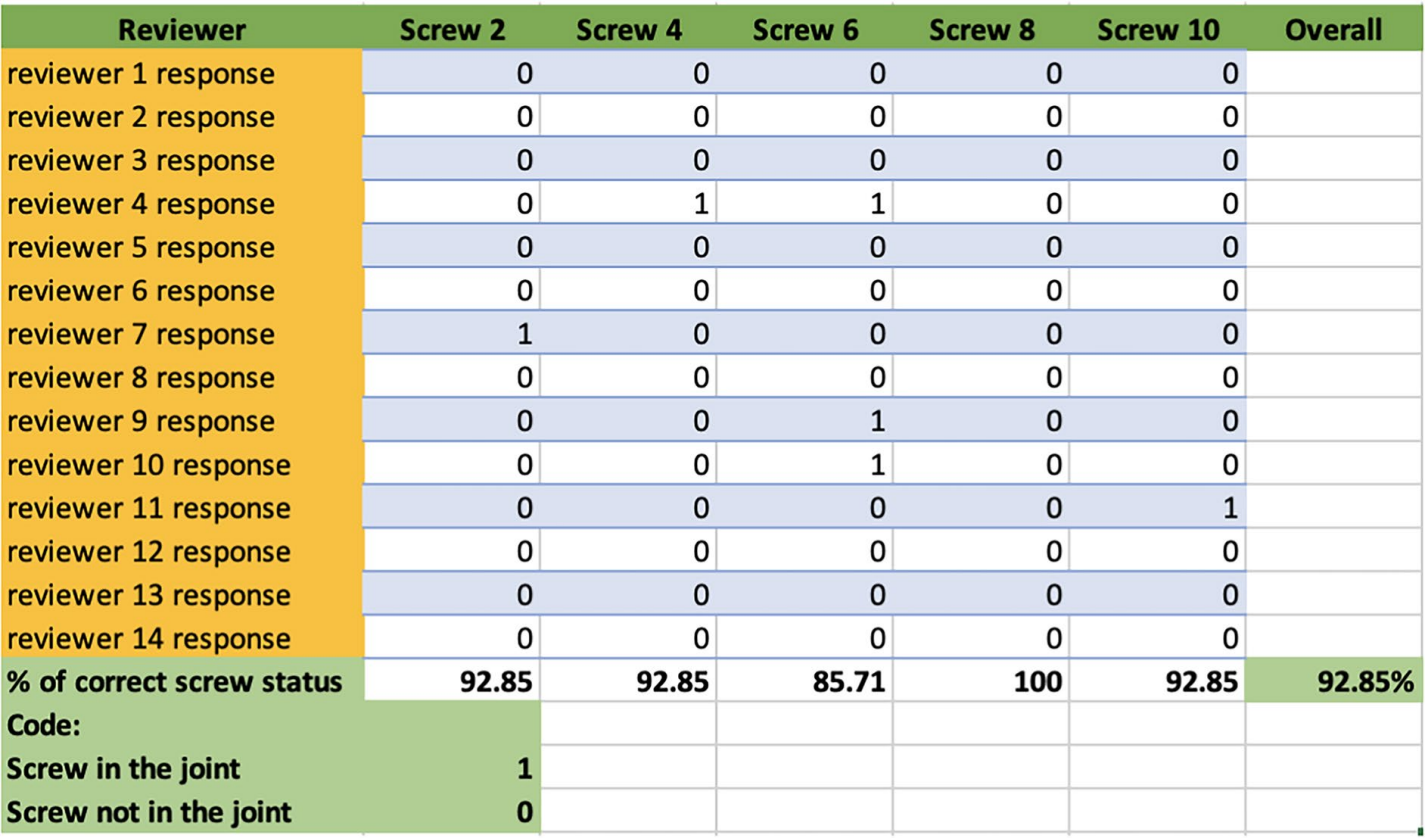
Reviewers’ response on the extra-articular screws’ positions

### Intra-articular screw position

Regarding the intra-articular screws, the observers exhibited an overall accuracy rate of 98.57% (*n* = 69). The accuracy rate was 100% screw numbers 1, 3, 5, and 7. However, screw number 9 had an accuracy rate of 92.85%. All views were required by the observers to determine the presence of intra-articular penetration (Table 3).

**Table 3:**
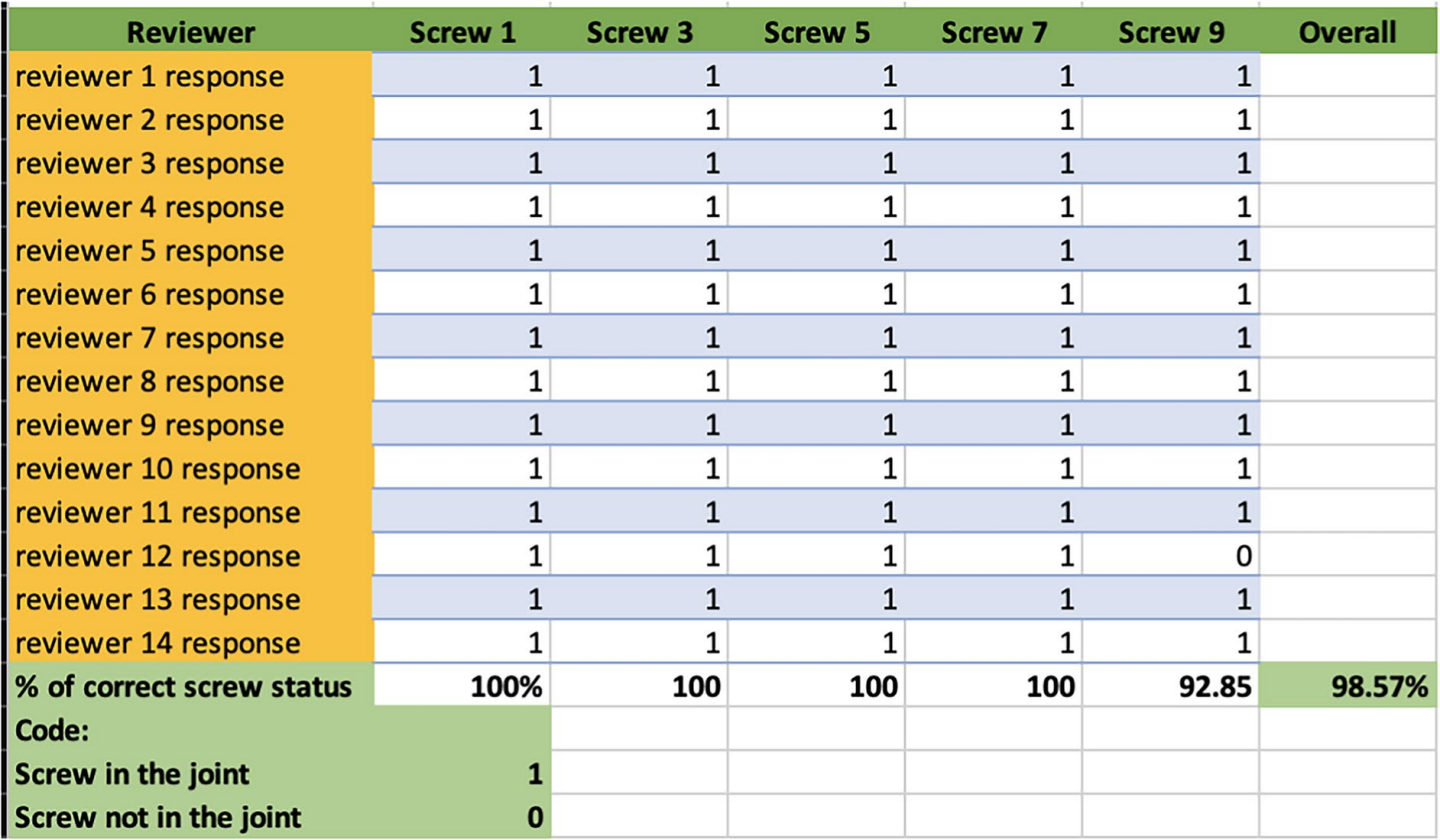
Reviewers’ response on the intra-articular screws’ positions

### Sensitivity, specificity, accuracy, and reliability

When evaluating screw position in relation to the hip joint, the overall accuracy rate for both surveys was 100%. The accuracy rate of distinguishing intra- and extra-articular screws was 82.1% for the anterior part of the anterior column and the superior part of the posterior column, 89.3% for the posterior part of the anterior column and the inferior part of the posterior column, and 92.9% for the quadrilateral plate. The interrater sensitivity was 100% for the anterior and posterior parts of the anterior column and the inferior part of the posterior column. However, the sensitivity was 87.5% for the superior part of the posterior column and 85.7% for the quadrilateral plate.

On the other hand, the interrater specificity was 100% for the quadrilateral plate and 80% for the superior part of the posterior column and the posterior part of the anterior column. However, the specificity was 78.6% for the inferior part of the posterior column and 66.7% for the anterior part of the anterior column (Table 4 summarizes the sensitivity, specificity of screw position, predictive values (positive predictive value (PPV) and negative predictive value (NPV)) and accuracy for the detection of intra-articular screw penetration using radiographs).

**Table 4:** Accuracy parameters for the detection of joint violation using radiographs

	Anterior part of anterior column	Posterior part of anterior column	Superior part of posterior column	Inferior part of posterior column	Quadrilateral plate
Sensitivity	100.0 (75.29–100)	100.0 (75.3–100.0)	87.5 (47.4–99.7)	100.0 (76.8–100.0)	85.7 (57.2–98.2)
Specificity	66.7 (38.4–88.2)	80.0 (51.9–95.7)	80.0 (56.3–94.3)	78.6 (49.2–95.3)	100.0 (76.8–100.0)
NPV	100.0	100.0	94.1 (71.6–99.0)	100.0	87.5 (66.0–96.2)
PPV	72.2 (56.0–84.2)	81.3 (61.2–92.3)	63.6 (41.2–81.4)	82.4 (63.1–92.7)	100.0
Correct interpretation (accuracy)	82.1(63.1–93.9)	89.3(71.8–97.7)	82.1(63.1–93.9)	89.3(71.8–97.7)	92.9(76.5–99.1)
Interrater Kappa coefficient	0.65 (0.39–0.91)	0.78 (0.6–1.0)	0.61 (0.31–0.91)	0.79 (0.56–1.0)	0.86 (0.67–1.0)
Intra-rater Kappa coefficient	0.82 (0.71–0.92)	0.89 (0.78–1)	0.78 (0.63–0.92)	0.89 (0.78–1)	0.92 (0.92–0.93)
*P* value for Kappa coefficient	< 0.01	< 0.01	< 0.01	< 0.01	< 0.01

There was a moderate interrater agreement for the anterior part of the anterior column (0.65) and superior part of the posterior column (0.61). There was substantial agreement for the posterior part of the anterior column (0.78) and inferior part of the posterior column (0.79) with a statistically significant *P*-value of < 0.01. However, an outstanding agreement was observed for the quadrilateral plate (0.86) with a statistically significant *P*-value of < 0.01. Moreover, there was an outstanding intra-rater agreement for all parts of the acetabulum (Table 4).

The overall interobserver and intra-observer agreements were measured using intraclass correlation coefficient (ICC), which was found to be 0.709 and 0.86, respectively. This indicates a strong agreement on screw position between the ratings made by different reviewers based on radiographs readings.

Further analysis was conducted to identify the most effective radiographic view to verify joint clearance for each screw position. It was evident that the utilization of the obturator outlet view had accuracy rates of 78.5% and 61.5% in determining joint clearance in the anterior and posterior parts of the anterior column, respectively. For the iliac oblique view and iliac inlet views, 63.6% and 78.5% of the reviewers were accurate in identifying joint clearance in the superior and inferior parts of the posterior column, respectively. The accuracy rate was 38.5% when considering screw positions in the quadrilateral plate. Significant disparities were detected in the responders’ distribution of the ideal radiographic for the assessment of joint clearance (*P* < 0.001) (Table 5).

**Table 5:** Screws positions with the corresponding best views to confirm joint clearance

Screws name	Joint violation	Screw position	Views used by responders to judge screw position
Screw 1	Yes	Anterior part of anterior column	Obturator oblique 71.4% Obturator inlet 57.1% Obturator outlet 78.6%
Screw 2	No		
Screw 3	Yes	Posterior part of anterior column	Obturator oblique 38.5% Obturator outlet 61.5%
Screw 4	No		
Screw 5	Yes	Superior part of posterior column	Iliac oblique 63.6%
Screw 6	No		
Screw 7	Yes	Inferior part of posterior column	Iliac oblique 57% Iliac inlet 78.6% Iliac outlet 71.4%
Screw 8	No		
Screw 9	Yes	Quadrilateral plate	Pelvis AP 38.46%
Screw 10	No		

## Discussion

Intra-articular screw placement is considered a major complication of acetabular surgery. Ensuring the absence of hip joint violation is crucial, and the radiographic assessment such screws presents a substantial challenge when evaluating acetabular fixation [15, 16].

This study revealed that in cases where a screw violates the joint, all seven radiographic views consistently show the screw, or part of it, within the joint space. This demonstrated a relatively high accuracy and agreement among the reviewers. It should be noted that a single radiographic view might be sufficient to dismiss the possibility of joint violation. A challenge arises when screws are positioned in close proximity to the joint during acetabular fixation. This is particularly true as certain views might indicate joint, even when it is not. Therefore, when dealing with concave surfaces, it is crucial to use a single specific view to guarantee joint clearance. Screw number 10, positioned outside the joint but in close proximity to the medial wall of the acetabulum, was shown violating the joint in all radiographic views except a single one, the anteroposterior view.

This study confirms the hypothesis that when placing a screw aiming towards a concave articular surface, a single fluoroscopic view is needed to establish its extra-articular placement. Based on previous studies, the acetabulum is conceptualized as a sphere, while the screw is viewed as a line. When the line and sphere intersect, it is challenging to identify a projection angle that visually separates them. On the other hand, when the sphere and line do not intersect, at least one view will clearly show that they are separated, despite some projections indicating possible penetration [16, 17]. However, resorting to repeated fluoroscopies to locate the intersected direction is not desirable, and a precise acetabular view depending on screw’s location is recommended to reduce radiation exposure.

By viewing the acetabulum as a sphere and the screw as a linear object, the fluoroscope can identify an imaging projection where the acetabulum and screw are distinct from each other. Occasionally, when observing through a sphere, the screw might seemingly reside within the sphere, even if it is not the case. However, if any single view demonstrated a clear demarcation between the sphere (acetabulum) and the screw, it signifies their lack of contact [18].

Understanding pelvic AP, oblique and combined views increased the accuracy of evaluating screws’ trajectories and increased the safety of acetabular fixation [19–21]. The findings of this study can be reflected in clinical practice by providing a better judgment on acetabular screw penetration and reducing the radiological exposure.

Several studies have described the ideal views to rule out peri-acetabular screw penetrations [15, 16, 22–30]. In their retrospective review, Lim et al. used postoperative CT scans to assess infra-acetabular screw penetration in posterior wall fixation of the acetabulum. Their findings indicated that the outlet view is the most reliable view to confirm joint clearance [15]. This type of screw corresponds to screw numbers 9 and 10 in this study. The results of this study showed that AP view could reliably confirm acetabular clearance, although the outlet view was not included in this investigation. As this study is based on anatomical models and real-time experiments, our findings are more consistent with the acetabular anatomy and easier to perform intra-operatively, especially when performing the surgeries in lateral position. Nevertheless, Lin et al.’s anatomical specimen study added the lateral acetabular view to the conventional views in evaluating posterior wall fixation [16]. While this addition could be a valuable during surgery, the findings of this study showed that a single iliac oblique view can be sufficient.

Regarding the evaluation of intra-articular screw penetration in the anterior column and wall, it was evident that surgeons exhibited a higher accuracy rate in identifying joint clearance utilizing the obturator oblique (71.4% accuracy) and obturator oblique/outlet views (78.5% accuracy). These findings are consistent with previous reports in the literature [8, 16]. It is recommended to avoid utilizing the iliac oblique and iliac oblique/outlet or inlet views when evaluating the joint in anterior column screws. As reported by Norris et al. [17], interpretation of these views in isolation superimposes the screw trajectory with the hip joint, creating the illusion of joint violation regardless of its actual position in relation to the acetabular dome. Furthermore, the responses associated with the iliac oblique and iliac oblique/outlet views demonstrated a considerable level of uncertainty.

Regarding the evaluation of intra-articular screw penetration in the posterior column and wall, surgeons demonstrated the highest rate of accuracy in evaluating joint clearance utilizing the iliac oblique (63.6% accuracy) and iliac oblique/inlet views (78.5% accuracy).

Carmack et al. determined in a cadaveric study that fluoroscopy and computed tomography share equal accuracy in identifying intra-articular screw penetration [23]. They further recommended revising posterior acetabular wall screws when intraoperative fluoroscopic imaging does not confirm extra-articular placement. In a separate study by Rashidifard et al., the positioning of lag screw fixation in posterior wall acetabulum fractures was evaluated [24]. By comparing intraoperative fluoroscopic views—melding iliac oblique with either inlet or outlet tilt—with postoperative CT imaging, they determined that the fluoroscopic results were consistent with CT findings. This consistency was particularly notable when screws were located less than 5 mm from the articular surface. Consequently, Rashidifard et al. asserted that intraoperative fluoroscopy is a dependable technique for the accurate placement of posterior wall lag screws, minimizing the likelihood of intra-articular screw misplacement.

### Limitations

There are several limitations to acknowledge in this study. Firstly, as the study was conducted on pelvic models, the generalizability of the findings to real-life scenarios might be restricted. Secondly, the results obtained are based on a normally shaped acetabulum, necessitating caution when dealing with anatomical variations. Thirdly, the impact of soft tissue on image quality and radiographic penetration was not accounted for. Additionally, the models did not include fractures, as well as any extra plates and screws, which could potentially affect the surgeon’s interpretation of fluoroscopic images.

## Conclusion

This study confirms the hypothesis that in a concave surface/joint fixation, such as the acetabulum, the probability of joint violation is unlikely if there is no evidence of it within a single fluoroscopic view. In acetabulum fracture fixation with a screw violating the joint, the screw’s presence was evident within the joint space in all fluoroscopic views. However, the absence of joint violation in one fluoroscopic view was adequate to rule out joint penetration.
